# Lifetime risk of cardiovascular-renal disease in type 2 diabetes: a population-based study in 473,399 individuals

**DOI:** 10.1186/s12916-022-02234-2

**Published:** 2022-02-07

**Authors:** Ruiqi Zhang, Jil Billy Mamza, Tamsin Morris, George Godfrey, Folkert W. Asselbergs, Spiros Denaxas, Harry Hemingway, Amitava Banerjee

**Affiliations:** 1 Robertson Centre for Biostatistics, Institute of Health and Wellbeing, University of Glasgow, Glasgow, UK; 2 Medical and Scientific Affairs, BioPharmaceuticals Medical, AstraZeneca, Cambridge, UK; 3 Institute of Health Informatics, University College London, London, UK; 4 Department of Cardiology, Division Heart & Lungs, University Medical Center Utrecht, Utrecht University, Utrecht, The Netherlands; 5 Institute of Cardiovascular Science, Faculty of Population Health Sciences, University College London, London, UK; 6 Health Data Research UK London, University College London, London, UK; 7 Barts Health NHS Trust, London, UK; 8 University College London Hospitals NHS Trust, London, UK

**Keywords:** Cardiovascular, Kidney, Type 2 diabetes, Lifetime, Population health, Attributable risk

## Abstract

**Background:**

Cardiovascular and renal diseases (CVRD) are major causes of mortality in individuals with type 2 diabetes (T2D). Studies of lifetime risk have neither considered all CVRD together nor the relative contribution of major risk factors to combined disease burden.

**Methods:**

In a population-based cohort study using national electronic health records, we studied 473,399 individuals with T2D in England 2007–2018. Lifetime risk of individual and combined major adverse renal cardiovascular events, MARCE (including CV death and CVRD: heart failure; chronic kidney disease; myocardial infarction; stroke or peripheral artery disease), were estimated, accounting for baseline CVRD status and competing risk of death. We calculated population attributable risk for individual CVRD components. Ideal cardiovascular health was defined by blood pressure, cholesterol, glucose, smoking, physical activity, diet, and body mass index (i.e. modifiable risk factors).

**Results:**

In individuals with T2D, lifetime risk of MARCE was 80% in those free from CVRD and was 97%, 93%, 98%, 89% and 91% in individuals with heart failure, chronic kidney disease, myocardial infarction, stroke and peripheral arterial disease, respectively at baseline. Among CVRD-free individuals, lifetime risk of chronic kidney disease was highest (54%), followed by CV death (41%), heart failure (29%), stroke (20%), myocardial infarction (19%) and peripheral arterial disease (9%). In those with HF only, 75% of MARCE after index T2D can be attributed to HF after adjusting for age, gender, and comorbidities. Compared with those with > 1, < 3 and ≥3 modifiable health risk behaviours, achieving ideal cardiovascular health could reduce MARCE by approximately 41.5%, 23.6% and 17.2%, respectively, in the T2D population.

**Conclusions:**

Four out of five individuals with T2D free from CVRD, and nearly all those with history of CVRD, will develop MARCE over their lifetime. Early preventive measures in T2D patients are clinical, public health and policy priorities.

**Supplementary Information:**

The online version contains supplementary material available at 10.1186/s12916-022-02234-2.

## Background

High fasting plasma glucose, which constitutes diabetes mellitus, ranks only behind high blood pressure and smoking (6.53 million [5.23–8.23] deaths and 171 million [144–201] DALYs) as a cause of global burden of disease [1]. There are an estimated 451 million people living with diabetes worldwide, increasing to 693 million by 2045 [2], with an annual cost US$1.31 trillion (95% CI 1.28–1.36) or 1.8% (95% CI 1.8–1.9) of global gross domestic product (GDP) [3]. Type 2 diabetes (T2D) accounts for more than 90% of individuals with diabetes [4]. A global approach to the prevention of this disease burden requires knowledge across different components of the long-term health risk associated with T2D.

Associations between T2D and increased risk of cardiovascular diseases (CVD) and chronic kidney disease (CKD) are well-defined with international consensus guideline recommendations regarding preventive management [5, 6]. However, these diseases have been mainly studied in isolation rather than together, despite significant clinical overlap and frequent multi-morbidity. Whether renin-angiotensin system-blocking agents [7] or the novel sodium-glucose cotransporter-2 (SGLT2) inhibitors [8], pharmaceutical advances underline the close inter-relationships between the cardiovascular and renal systems. There is relevance to the current coronavirus (COVID-19) pandemic, where CVD, CKD, T2D and multi-morbidity are known to be associated with increased risk of infection and severe outcomes [9, 10], but the interplay of CVD, CKD and T2D is poorly characterised. Better understanding of how these diseases cluster over the life course [11] is required in order to inform their management and prevention during and beyond the pandemic [12].

Most people considered to be at low risk for morbidity and mortality (including CKD and CVD) in the short term are actually at high risk over their life course [13]. Estimates of lifetime risk of diseases allow a more holistic evaluation of the overall burden of a given disease in the general population, now and in the future, because they take into account both the risk of disease and competing risks (e.g. death from other diseases) until individuals reach old age. Lifetime risk of individual CVDs [13, 14] and CKD [15] has been estimated. Moreover, it has been shown that people with low risk factor burden have a substantially lower lifetime risk of CVD. Study of the epidemiology of the co-occurrence of CVD and CKD, and their relative contribution to overall disease burden could inform knowledge of disease progression, risk prediction, as well as prevention, management and health policy for T2D.

## Methods

The two main aims of this study are to investigate (1) the lifetime risk of composite and individual components of major adverse cardiovascular and renal events and (2) the population attributable risk of cardiovascular and renal disease (CVRD) in individuals with T2D.

### Data source

We used electronic health records (EHR) across primary care (Clinical Practice Research Datalink, CPRD-Aurum), hospital admissions (Hospital Episodes Statistics, HES), and death registry (Office for National Statistics, ONS) with prospective recording and follow-up; linked using unique national healthcare identifiers (CPRD and NHS Digital) [16]. Over 99% of England’s population is general practice (GP)-registered [1]. CPRD is representative by socio-demography, ethnicity and mortality.

### Study population

**Fig. 1:**
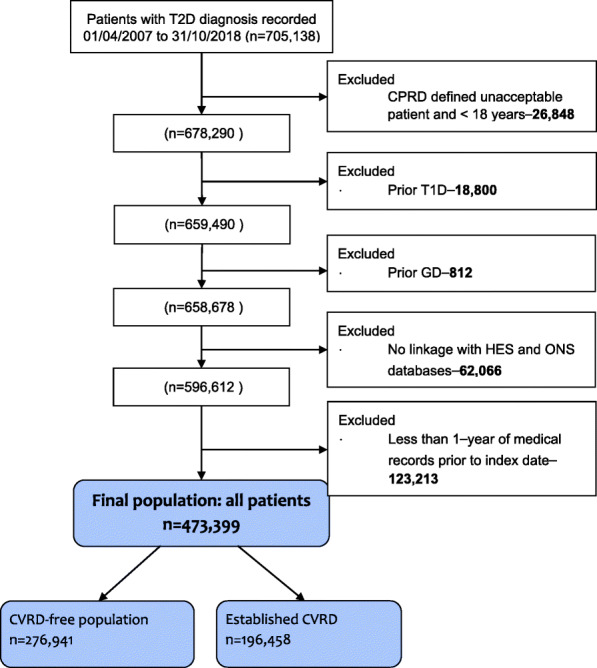
Study population: individuals with T2D and underlying cardiovascular and chronic kidney disease. Abbreviations: cardiovascular and renal diseases, CVRD; clinical practice research datalink, CPRD; gestational diabetes, GD; Type 1 diabetes, T1D, Hospital Episode Statistics, HES; office for national Statistics, ONS

We included individuals ≥18 years of age who had a record of T2D diagnosis for the first time between 1 April 2007 and 31 October 2018 and were registered with a general practice during the same period with at least one year of data prior to index date (1 Apr 2007). We excluded individuals with a general practice record of type 1 diabetes or gestational diabetes prior to index date (Fig. 1). Follow-up was from day after index date until the earliest of study outcome, death, transfer out of practice, or study end date (31 October 2018). Baseline comorbidities (any diagnosis record), medications (within 12 months) and investigations (latest) were searched in prescribed drugs, hospital records and general practice records prior to index T2D diagnosis, consistent with our prior analysis [1]. Specifically, baseline CVRD status was identified in primary care using Snomed codes (MedcodeIDs) for diagnoses and symptoms, as well as using ICD10 codes from hospitalisations. Outcomes were identified using ICD10 codes for in-patient hospitalisations listed as a diagnosis at any position. A complete list of definitions is available in Additional file 1: Table S1 and Additional file 17: Table S2, respectively. Data were analysed based on observed cases only without imputing of missing values, while those without the presence of diagnostic codes—prescription records of interest were assumed to be disease free—have not taken the drug.

### Lifetime risk

For lifetime risk analysis, a subset of individuals aged 45–99 years at index date were included. Baseline CVRD was defined by history of angina; myocardial infarction, MI; heart failure, HF; stroke/transient ischaemic attack, TIA; peripheral artery disease, PAD; chronic kidney disease, CKD; or prescription for nitrates. The CVRD-free population was the reference group, and those with only one co-morbid CVRD were also looked at. Thus, the study population was stratified into mutually exclusive categories based on baseline CVRD: (1) CVRD-free; and only (2) HF; (3) CKD; (4) MI; (5) stroke; or (6) PAD.

### Attributable risk among the exposed and population attributable risk

The population attributable risk analysis was conducted in the overall study population. The population were stratified into high (exposed) or low risk (unexposed) groups for each baseline risk factor, as well as an overall cardiovascular health indicator.

### Outcomes

The primary outcome is major adverse renal cardiovascular events (MARCE). MARCE were defined as a composite of cardiovascular (CV) death, and hospitalisations due to CVRD: including HF, CKD, MI, stroke or PAD. Secondary outcomes are the individual components of MARCE. In sensitivity analyses, we assessed major adverse cardiovascular events (MACE), defined as MI, stroke or CV death. We used validated EHR phenotyping algorithms and tools for national structured data [16].

### Analysis

#### Lifetime risk

We estimated lifetime risk (i.e. risk of developing disease over remaining years of life) for CVD, CKD and composite outcomes using published methodology [13] in those with T2D. Briefly, it is a modified technique of survival analysis, adjusting for competing risk of death from other causes (instead of treating subjects who die as censored observations in traditional survival analysis). In our analysis, participants contributed information on incidence of disease and death free of disease for each age attained during follow-up. For each study population based on baseline CVRD status, remaining lifetime risks were calculated for the first occurrence of specific CVRD events at 45–99 years of age, with death or death free of CVRD as a competing event. For the reference CVRD-free population, lifetime risk for estimated for men and women at different age groups (55, 65, 75, and 85 years of age).

#### Attributable risk among the exposed and population attributable risk

Attributable risk among the exposed (ARE) assesses excess risk among those exposed (or at high risk)—how much of an outcome may be attributable to a risk factor (i.e. an estimate of the excess risk) in a population exposed to that factor. We fitted age, sex and baseline cardio-renal status adjusted logistic models to estimate predicted probabilities of MARCE and component events, comparing patients with and without risk factors. The adjusted estimates of outcome risk in the exposed (or high-risk) and unexposed (low-risk) groups were then used to obtain the excess attributable risk of CVRD comorbidities and other risk factors: ARE *=* (*R*_*e*_ *− R*_*u*_)/*R*_*e*_, where *R*_*e*_ is the estimated risk of the outcome in the exposed and *R*_*u*_ is the estimated risk in the unexposed groups.

Using ARE, we estimated population attributable risk (PAR), the proportion of incidence of a disease in the population (exposed and unexposed) due to exposure, for CVD, CKD and composite outcomes: PAR=ARE*proportion of exposed in the population (*P*_*e*_)*.* The American Heart Association has defined “ideal cardiovascular health” and “Life’s Simple 7” (health factors: blood pressure, cholesterol, and glucose; and health behaviours: cigarette smoking, physical activity, diet, and body mass index) [18]. We estimated the reduction in CVRD and mortality in individuals with T2D by achieving ideal cardiovascular health (with the exception of diet which was not recorded in the available data). Analyses were in SAS Enterprise Guide V7.15.

## Results

### Baseline characteristics at incident T2D diagnosis

The study population included 473,399 individuals with incident T2D: mean age 63.5 years (SD 14.4), 45.7% female, median follow-up 6 years [IQR 3–9], and 59% (*n*=276,941) CVRD-free, i.e. no known history of MI, HF, stroke/TIA, PAD, AKI, CKD, or prescription for nitrates. Individuals with no history of CVRD were younger (mean 58.3 years, SD 13.7) than those with history of HF (mean 70.2, S.D. 13.4), CKD (mean 71.8, S.D. 12.2), MI (mean 67.3, S.D. 12.4), stroke (mean 69.3, S.D. 12.6) and PAD (68.4, S.D.11.1). There were significant differences by gender: 46.4% female for those without history of CVRD, compared with HF: 47.9%, CKD: 58.3%, MI: 29.9%, stroke: 45.5% and PAD: 36.4% (Table 1).

**Table 1:** Baseline characteristics in individuals with type 2 diabetes and pre-existing cardiovascular disease and chronic kidney disease

		All T2D	CVRD free	HF only	CKD only	MI only	Stroke only	PAD only	Multiple CVRD
		473,399	276,941	5310	35,019	2721	17,044	5898	126,615
Follow-up (years)	Median [IQR]	6 [3 , 9]	6 [3 , 10]	4 [2 , 8]	5 [3 , 9]	6 [3 , 10]	5 [3 , 9]	6 [3 , 10]	5 [3 , 9]
Age (years)	Mean (SD)	63.5 (14.4)	58.3 (13.7)	70.2 (13.4)	71.8 (12.2)	67.3 (12.4)	69.3 (12.6)	68.4 (11.1)	70.9 (11.71)
Female	N (%)	216,273 (45.7%)	128,516 (46.4%)	2542 (47.9%)	20,411 (58.3%)	813 (29.9%)	7760 (45.5%)	2147 (36.4%)	51,891 (41.0%)
BMI	Mean (SD)	31.5 (6.9)	32.1 (7.0)	32.7 (8.1)	30.8 (6.7)	30.6 (6.2)	30.2 (6.4)	29.9 (6.3)	30.7 (6.40)
Smoking status	Never	69,360 (19.7%)	40,209 (20.5%)	765 (19.1%)	6178 (23.4%)	326 (16.3%)	2333 (18.3%)	633 (12.4%)	18,348 (17.9%)
	Former	162,659 (46.3%)	82,255 (42.0%)	2083 (51.9%)	12,539 (47.5%)	1054 (52.8%)	6056 (47.6%)	2364 (46.3%)	54,739 (53.3%)
	Current	119,532 (34.0%)	73,296 (37.4%)	1162 (29.0%)	7658 (29.0%)	618 (30.9%)	4325 (34.0%)	2109 (41.3%)	29,675 (28.9%)
HbA1c Category	< 7%	131,242 (50.8%)	66,677 (48.4%)	1471 (51.9%)	12,527 (55.8%)	731 (50.0%)	4931 (54.4%)	1757 (46.8%)	41,950 (53.1%)
	7-10%	105,662 (40.9%)	56,517 (41.1%)	1148 (40.5%)	8750 (39.0%)	627 (42.9%)	3557 (39.3%)	1683 (44.8%)	32,490 (41.1%)
	> 10%	21,574 (8.3%)	14,451 (10.5%)	213 (7.5%)	1161 (5.2%)	103 (7.0%)	570 (6.3%)	313 (8.3%)	4604 (5.8%)
SBP (mmHg)	< 130	142,824 (32.2%)	79,050 (30.8%)	1944 (39.1%)	9947 (29.7%)	756 (31.7%)	4890 (31.0%)	1487 (26.7%)	43,201 (35.8%)
	130–150	224,531 (50.7%)	131,672 (51.3%)	2256 (45.3%)	17,648 (52.7%)	1241 (52.0%)	8191 (51.9%)	2919 (52.4%)	59,054 (48.9%)
	150+	75,840 (17.1%)	45,956 (17.9%)	775 (15.6%)	5894 (17.6%)	391 (16.4%)	2707 (17.1%)	1165 (20.9%)	18,437 (15.3%)
DBP (mmHg)	< 80	221,401 (50.0%)	106,627 (41.5%)	2858 (57.4%)	19,960 (59.6%)	1335 (55.9%)	8489 (53.8%)	3259 (58.5%)	76,401 (63.3%)
	80–100	206,329 (46.6%)	138,113 (53.8%)	1962 (39.4%)	12,820 (38.3%)	1012 (42.4%)	6899 (43.7%)	2205 (39.6%)	42,248 (35.0%)
	100+	15,412 (3.5%)	11,906 (4.6%)	156 (3.1%)	710 (2.1%)	42 (1.8%)	400 (2.5%)	107 (1.9%)	2019 (1.7%)
Atrial fibrillation	*N* (%)	32,972 (7.0%)	6392 (2.3%)	1884 (35.5%)	2321 (6.6%)	206 (7.6%)	1739 (10.2%)	354 (6.0%)	18,569 (14.7%)
HDL (mmol/L)	Mean (SD)	1.2 (0.37)	1.2 (0.36)	1.3 (0.38)	1.3 (0.39)	1.2 (0.35)	1.3 (0.38)	1.2 (0.37)	1.2 (0.37)
LDL (mmol/L)	Mean (SD)	2.7 (1.03)	2.9 (1.02)	2.6 (0.94)	2.6 (0.99)	2.4 (0.96)	2.4 (0.95)	2.5 (1.02)	2.3 (0.94)
Triglyceride (mmol/L)	Median (IQR)	1.7 [1.2 , 2.4]	1.8 [1.2 , 2.5]	1.6 [1.1 , 2.3]	1.7 [1.2 , 2.3]	1.6 [1.1 , 2.4]	1.6 [1.1 , 2.3]	1.7 [1.2 , 2.5]	2.0 (1.29)
Physical activity	Inactive	83,736 (43.0%)	39,805 (36.8%)	1083 (52.1%)	6565 (47.4%)	388 (37.7%)	3424 (50.6%)	1166 (49.8%)	30,344 (51.5%)
	Moderately inactive	17,637 (9.1%)	11,148 (10.3%)	193 (9.3%)	1272 (9.2%)	84 (8.2%)	530 (7.8%)	149 (6.4%)	4144 (7.0%)
	Moderately active	70,060 (36.0%)	41,557 (38.4%)	606 (29.1%)	4502 (32.5%)	451 (43.8%)	2159 (31.9%)	798 (34.1%)	19,655 (33.4%)
	Active	23,315 (12.0%)	15,750 (14.5%)	198 (9.5%)	1503 (10.9%)	106 (10.3%)	655 (9.7%)	227 (9.7%)	4787 (8.1%)
**CVD risk treatment**									
Low dose aspirin		29.0%	14.2%	25.1%	27.5%	50.2%	45.1%	49.8%	58.4%
Statins		53.1%	40.4%	48.7%	58.9%	64.2%	68.5%	69.5%	76.5%
Anti-hypertensive medications:		55.6%	43.1%	73.2%	73.0%	59.5%	63.9%	62.2%	75.5%
ACE inhibitors		34.7%	26.0%	51.7%	44.2%	40.9%	39.5%	38.7%	48.9%
ARBs		13.1%	9.3%	17.7%	20.9%	13.0%	13.2%	12.6%	18.7%
Calcium channel blockers		26.3%	20.1%	25.0%	35.9%	22.6%	33.4%	33.2%	36.0%
Beta blockers		23.0%	11.1%	41.0%	23.3%	40.1%	19.8%	13.7%	48.2%
Potassium-sparing diuretics		3.6%	1.2%	19.4%	3.5%	2.5%	2.1%	2.1%	7.8%
Loop diuretics		12.7%	4.2%	55.5%	15.6%	9.5%	10.1%	9.7%	27.6%
Thiazides		13.9%	12.5%	10.8%	22.7%	11.9%	18.5%	18.4%	14.0%
Warfarin		5.2%	1.7%	26.0%	4.9%	5.7%	7.6%	6.7%	10.9%
P2Y12 inhibitors		4.8%	0.4%	1.5%	1.1%	7.0%	14.8%	8.3%	14.1%
Other antiplatelets		26.2%	12.7%	22.4%	24.7%	45.2%	42.5%	44.4%	53.1%
Corticosteroids		19.9%	16.8%	25.6%	22.2%	15.6%	20.4%	20.6%	25.4%
Any CVD risk treatment		71.4%	59.6%	83.9%	84.8%	76.5%	83.7%	84.7%	90.0%
**Glucose-lowering treatment**									
SGLT-2 inhibitors		0.2%	0.2%	0.2%	0.2%	0.5%	0.1%	0.3%	0.2%
Metformin		39.4%	39.8%	36.3%	38.9%	39.6%	38.1%	45.6%	39.0%
Sulfonylurea		15.5%	12.6%	15.7%	22.0%	16.1%	16.1%	22.1%	19.5%
DPP-4 inhibitors		1.9%	1.6%	1.6%	2.9%	1.8%	1.5%	1.8%	2.2%
GLP-1RA		0.5%	0.5%	0.7%	0.5%	0.6%	0.3%	0.7%	0.5%
Metiglinides		0.3%	0.2%	0.1%	0.3%	0.3%	0.3%	0.4%	0.3%
Glitazones		4.9%	4.5%	3.5%	7.0%	5.3%	4.2%	7.5%	5.3%
Acarbose		0.2%	0.1%	0.2%	0.3%	0.2%	0.2%	0.4%	0.3%
Insulins		7.2%	4.8%	5.9%	10.5%	7.3%	6.0%	12.1%	11.3%

Abbreviations: *ACE* angiotensin-converting enzyme, *ARBs* angiotensin II receptor blockers, *BMI* body mass index, *CVD* cardiovascular disease, *DBP* diastolic blood pressure, *DPP-4* dipeptidyl peptidase-4, *GLP-1RA* glucagon-like peptide-1 receptor antagonist, *HbA1c* glycated haemoglobin, *HF* heart failure, *HDL* high-density lipoprotein, *CKD* chronic kidney disease, *CVD* cardiovascular disease, *HDL* low-density lipoprotein, *MI* myocardial infarction, *MARCE* major adverse renal cardiovascular events, *PAD* peripheral artery disease, *P2Y12* platelet adenosine diphosphate receptor, *SGLT2* sodium-glucose transport protein-2, *SBP* systolic blood pressure

Individuals without a history of CVRD had the lowest rates of atrial fibrillation (2.3%) and former smoking (42.0%), and the highest rate of HbA1c levels > 10% (10.5%). Blood pressure, cholesterol and triglycerides were similar across individuals, regardless of prior CVRD. Individuals without a history of CVRD had the lowest use of aspirin (14.2%), statins (40.4%), anti-hypertensive therapy (43.1%) and CVD risk-lowering therapy (59.6%) in the 12 months prior to index T2D diagnosis. However, there were gaps in evidence-based therapy for all patient groups, e.g. among those with prior MI, stroke and PAD, only 64.2%, 68.5% and 69.5% were taking statin, and 50.2%, 45.1% and 49.8% were taking aspirin. Use of glucose-lowering therapies was highest in those with PAD and lowest in those without prior CVRD (Table 1).

### Lifetime risk

**Fig. 2:**
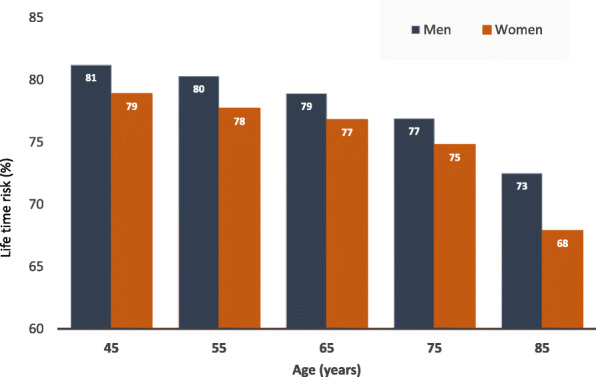
Lifetime risk of major adverse renal and cardiovascular events (MARCE) by gender for reference CVRD-free population. Major adverse renal cardiovascular events (MARCE) includes a composite of cardiovascular death, and any hospitalisations due to heart failure chronic kidney disease, myocardial infarction, stroke or peripheral artery disease

In individuals free from CVRD at age 45 years, lifetime risk of MARCE was 81% in men and 79% in women. Because remaining life expectancy and competing risks of death are considered, lifetime risk decreases with increasing age but remained high with increasing age, e.g. 77% and 75% at age 75 years, and 73% and 68% at age 85 years in men and women respectively (Fig. 2). In individuals with HF, CKD, MI, stroke and PAD, lifetime risk of MARCE was very high: 97.1% (95%CI 97.1-100.0), 93.3% (93.3–96.3), 97.6% (97.6–100.0), 89.2% (89.1–92.1) and 90.8% (90.8–98.9) respectively, compared with 80.0% (79.7–80.4) in those without CVRD (Table 2).

**Table 2:** Lifetime risk (%) with 95% confidence interval of individual and composite major adverse renal and cardiovascular events by baseline disease status in T2D

Baseline status	Outcomes						
	HF	CKD	MI	Stroke	PAD	CVD death	MARCE
Reference: CVRD free	29.2 (27.5–29.7)	53.6 (52.6–54.1)	18.9 (17.2–19.3)	20.2 (18.5–20.6)	9.2 (8.1–9.5)	40.5 (40.0–41.1)	80.0 (79.7–80.4)
HF only	–	66.0 (65.2–70.6)	25.7 (21.6–29.4)	18.7 (13.2–21.7)	12.8 (8.8–15.5)	66.0 (65.0–69.4)	97.1 (97.1–100)
CKD only	34.7 (32.9–36.2)	–	20.7 (18.7–22.0)	20.3 (18.2–21.6)	13.0 (11.2–14.3)	45.7 (44.3–47.2)	93.3 (93.3–96.3)
MI only	44.0 (37.2–49.8)	53.2 (49.1–58.8)	–	24.4 (15.9–28.1)	14.3 (9.1–17.9)	60.3 (56.1–64.4)	97.6 (97.6–100)
Stroke only	28.8 (25.5–30.5)	54.5 (52.9–56.7)	23.8 (20.4–25.8)	–	12.3 (10.2–13.6)	56.2 (54.7–57.9)	89.2 (89.1–92.1)
PAD only	35.2 (30.0–38.4)	56.0 (53.8–61.4)	29.2 (21.4–33.7)	27.7 (21.0–31.8)	–	52.5 (49.0–56.0)	90.8 (90.8–98.9)

Abbreviations: *HF* heart failure, *CKD* chronic kidney disease, *CVD* cardiovascular disease, *MI* myocardial infarction, *MARCE* major adverse renal cardiovascular events, *PAD* peripheral artery disease

Lifetime risk of HF was highest in those with MI (44.0%, 37.2–49.8) and PAD (35.2%, 30.0–38.4), compared with 29.2% (27.5–29.7) in those without CVRD. For CKD, lifetime risk was highest in those with HF (66.0%, 65.2–70.6), but similar across other categories; CVRD-free: 53.6% (52.6-54.1), MI: 53.2 (49.1–58.8), stroke: 54.5 (52.9–56.7) and PAD: 56.0 (53.8–61.4). Of the major renal and CVD events, the lifetime risk of PAD was lowest across patient groups; e.g. CVRD-free: 9.2% (8.1–9.5), HF: 12.8% (8.8–15.5), MI: 14.3% (9.1–17.9%).

Lifetime risk of MI and CV death varied by a history of pre-existing CVRD and were highest in those with HF and PAD. The lifetime risk of stroke was lowest in individuals with HF (18.7%, 13.2–21.7), compared with 20.2% (18.5–20.6) in individuals without history of CVRD (Fig. 2, Table 2). Conversely, lifetime risk of HF was lowest in those with stroke (28.8%, 25.5–30.5), compared with those without CVRD (29.2%, 27.5–29.7).

### Attributable risk among the exposed and population attributable risk

Out of the individual cardiovascular and renal comorbidities, ARE of MARCE was highest in T2D patients with HF at 0.748, i.e. in those with HF only, 75% of MARCE after index T2D can be attributed to HF. Nearly 5% of incident MARCE is attributable to CKD (PAR 0.046) and 2% (PAR 0.019) to stroke. The PAR is lower for HF compared to CKD due to fewer individuals with HF only (*n*=5310, 1.12%) compared to CKD (*n*=35,019, 7.40%), as PAR accounts for both strength of increased risk and prevalence of the risk factor. Among other modifiable risk factors, incidence of MARCE in severely obese T2D patients would decrease by 30% by modification to normal BMI. If the BMI of severely obese people is reduced to normal levels, 3% of MARCE in the T2D population could be prevented (PAR=0.031).

Smoking, poor HbA1c control (> 10%) and hypertension are risk factors for MARCE: odds ratio (95% CI) 1.15 (1.12–1.19), 1.96 (1.88–2.04) and 1.24 (1.21–1.27), respectively, after adjusting for age, gender and baseline CVRD status. Over a median of 6 years, current smoking, poor HbA1c control and hypertension conferred an additional 11.7%, 43.6% and 16.7% risk on MARCE. PAR estimates for the hypothetical modification of smoking, HbA1c or BP to reference categories are 4.0%, 3.6% and 2.8% for MARCE. Table 3 shows associations between risk factors and MARCE, and associated PAR.

**Table 3:** Age, sex and baseline CVRD status-adjusted risk estimates and population attributable fractions (PAR) for non-modifiable and modifiable risk factors of MARCE in type 2 diabetes

Risk factors	Category	OR (95%CI)	Estimated risk	ARE	% of events (Pe)	PAR
Non-modifiable						
Age	< 45	1	0.030		11.1	
	45–54	1.55 (1.49, 1.61)	0.045	0.345	17.5	0.060
	55–64	2.47 (2.38, 2.55)	0.070	0.577	23.4	0.135
	65–74	4.35 (4.20, 4.50)	0.118	0.747	24.3	0.181
	75–84	7.75 (7.47, 8.03)	0.192	0.845	17.8	0.150
	85+	9.79 (9.38, 10.21)	0.231	0.871	5.9	0.052
Sex	Female	1	0.514		45.7	
	Male	1.20 (1.18, 1.23)	0.559	0.082	54.3	0.044
Modifiable						
Comorbidities	CVRD free	1	0.045		58.5	
	HF only	4.60 (4.20, 5.04)	0.177	0.748	1.1	0.008
	CKD only	2.86 (2.76, 2.96)	0.118	0.621	7.4	0.046
	MI only	4.29 (3.79, 4.85)	0.167	0.733	0.6	0.004
	Stroke only	2.17 (2.06, 2.28)	0.092	0.515	3.6	0.019
	PAD only	3.81 (3.54, 4.10)	0.151	0.704	1.2	0.009
	Multiple CVRD	4.69 (4.58, 4.79)	0.180	0.751	27.6	0.207
BMI	Normal (20–24)	1	0.124		12.7	
	Under weight (< 20)	0.96 (0.88, 1.04)	0.120	-0.039	1.6	-0.001
	Over weight (25–29)	0.99 (0.96, 1.03)	0.123	-0.005	31.6	-0.002
	Obese (30–39)	1.12 (1.09, 1.16)	0.137	0.096	43.3	0.042
	Severely obese (40+)	1.49 (1.42, 1.56)	0.174	0.287	10.8	0.031
Smoking	Never	1	0.129		19.7	
	Former	1.03 (1.00, 1.06)	0.132	0.026	46.3	0.012
	Current	1.15 (1.12, 1.19)	0.146	0.117	34.0	0.040
HbA1c	< 7%	1	0.109		50.8	
	7–10%	1.45 (1.42, 1.48)	0.150	0.276	40.9	0.113
	10%	1.96 (1.88, 2.04)	0.192	0.436	8.3	0.036
Hypertension	SBP < 150 and DBP < 100	1	0.134		83.3	
	SBP > 150 or DBP > 100	1.24 (1.21, 1.27)	0.161	0.167	16.7	0.028
High cholesterol	HDL ≥1 and LDL ≤3 and triglyceride ≤2.3	1	0.127		38.4	
	HDL ≤1 or LDL ≥3 or triglyceride ≥2.3	1.10 (1.08, 1.13)	0.139	0.083	61.6	0.051
Physical activity	Inactive	2.19 (2.07, 2.32)	0.140	0.506	43.0	0.217
	Moderately inactive	1.01 (0.93, 1.09)	0.069	0.005	9.1	0.000
	Moderately active	1.49 (1.41, 1.58)	0.100	0.307	36.0	0.110
	Active	1	0.069		12.0	
Overall cardiovascular health*	Ideal	1	0.080		1.0	
	≥1 risk factor	1.84 (1.72, 1.97)	0.138	0.419	99.0	0.415

*Risk factors: BMI ≥30, current smoker, HbA1c ≥7%, hypertension, high cholesterol, inactive. Each multivariable logistic model adjusted for age, sex and baseline cardio-renal status. ARE *=* (*R*_*e*_ *− R*_*u*_)/*R*_*e*_, where *R*_*e*_ is the estimated risk of the outcome in the exposed and *R*_*u*_ is the estimated risk in the unexposed groups. PAR=ARE*proportion of exposed in the population (*P*_*e*_). *Abbreviations*: *ARE* attributable risk among the exposed, *BMI* body mass index, *CVRD* cardiovascular and renal diseases, *DBP* diastolic blood pressure, *HbA1c* glycated haemoglobin, *HF* heart failure, *HDL* high-density lipoprotein, *CKD* chronic kidney disease, *HDL* low-density lipoprotein, *MI* myocardial infarction, *SBP* systolic blood pressure, *PAR* population attributable risk, *PAD* peripheral artery disease

Compared with those with more than 1 modifiable health risk behaviours, achieving ideal cardiovascular health could substantially reduce MARCE in individuals with T2D by an estimated 41.5%. In those with ≥3 and < 3 modifiable health risk behaviours, achieving ideal cardiovascular health could prevent 17.2% and 23.6% of MARCE in the T2D population, respectively. Other CVRD outcomes are in Additional files Table S1-S9.

## Discussion

In the first nationally representative, population-based study of lifetime risk in individuals with T2D, we have four major findings. First, in newly diagnosed T2D, 4 out of 5 individuals without, and nearly all with, history of CVRD develop CVD or CKD events over their lifetime. Second, 1 in 2 individuals with newly diagnosed T2D develops CKD over their lifetime, increasing to 2 in 3 in those with HF. Third, ideal cardiovascular health in individuals with T2D could reduce the risk of CVRD and mortality in the entire T2D population by 37.1% and 46.3%, respectively. Fourth, evidence-based therapies are under-used for CVD risk reduction in individuals with T2D, regardless of underlying CVRD.

### Lifetime risk—a tool for lifelong prevention in diabetes

Prevention of particular microvascular and macrovascular complications is already a priority in high-risk individuals with T2D [5, 19]. More generally, there are increasing calls for prioritisation of non-communicable diseases and multi-morbidity during and after the pandemic, whether to reduce risk of severe COVID-19 or the overall global burden of disease [11, 3]. However, the majority of clinical and public health efforts rest on short-term risk, and even in prevention, 10 years is the maximum window over which risk is estimated and conveyed to patients. However, focus on acute and shorter-term outcomes under-estimates total disease burden, where nearly all individuals with T2D and previous CVRD go on and have further MARCE over their lifetime. Improved secondary prevention is a major opportunity to reduce healthcare costs to patients and health systems. Given high lifetime T2D risk globally [20–22], the high lifetime risk of CVRD which we describe, prevention earlier in the disease spectrum is of relevance to researchers, clinicians and policymakers, even in those with T2D alone (which is considered lower risk by existing prediction tools [23, 24]). For example, our findings may change the misperception of the risk status in the younger T2D population and inform change in national guidelines for diabetes and cardiorenal risk management in these individuals.

**Fig. 3:**
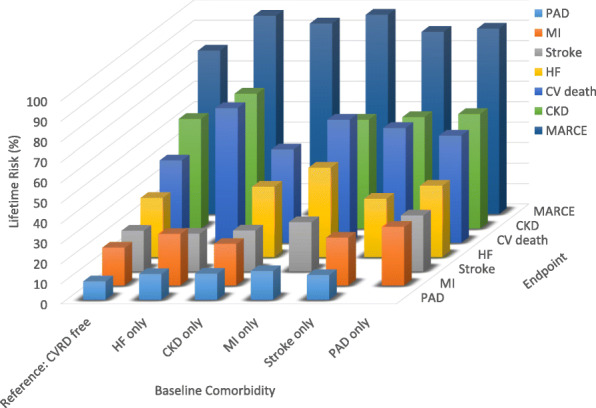
Lifetime risk of individual and composite major adverse renal and cardiovascular events. *Abbreviations*: cardiovascular and renal diseases, CVRD; heart failure, HF; chronic kidney disease, CKD; myocardial infarction, MI; peripheral artery disease, PAD

### Multi-morbidity over the life course

Compared with other diseases, those with T2D have a high lifetime risk of CKD (1 in 2), HF (2 out of 3) and CVRD (at least 8 out of 10). Research and practice in T2D and other long-term conditions emphasise multi-morbidity at baseline, but have neglected development of multi-morbidity over the life course [25], where benefits of primordial and primary prevention are greater for individuals, populations and health systems. The majority of scientific and clinical discourse has been disease-specific, with multi-morbidity focusing on number rather than specific combinations of diseases and risk factors. Studies have concentrated on coronary disease or CVD specifically [5]. Whether traditional statistical methods (e.g. regression analysis) or novel analytics such as clustering to discover subtypes or predict risk, studies have tended to consider individual diseases rather than groups of diseases together, such as CVRD [26]. Perhaps truly unsupervised approaches need to look at wider ranges of disease or composite exposures (e.g. CVRD) and outcomes (e.g. MARCE) over longer periods of time, if not over the life course, in order to more accurately reflect subtypes, distributions and trajectories of disease, with implications for chronic disease prevention and management.

### The need for integrated care in cardiovascular-renal disease

Cardiorenal [26] and cardiometabolic syndromes [5] have gained attention in recent years from diagnosis to treatment. We show a particular risk of CKD, suggesting future prioritisation for earlier preventive therapies. Our data support recognition of CVRD, MARCE and broader outcomes for clinical practice, public health and research, from trials to service delivery. A recent long-term study of individuals with HF in the UK showed high prevalence, hospitalisation and mortality in presence of T2D and CKD [27]. Median survival was lowest in individuals with HF, CKD and T2D (0.7–2.8 years depending on stage of CKD), compared with HF-T2D-only (4.1 years), HF-CKD only (2.2 years) and no CKD or HF (4.4 years). We now add importance of burden of HF in T2D, partly due to high lifetime risk (30%), but also because 75% of MARCE are attributable to HF in those with T2D and HF. Research and clinical guidelines in T2D have stressed atherosclerotic CVD, but must also emphasise primary and secondary prevention to CVD more broadly, particularly HF. Health systems have responded by specialisation, when greater integration of specialties, more emphasis on primary care, early identification, recognition of multi-organ impairment and treatment of the people at most risk at multiple stages in the lifelong trajectory of T2D are required. Joint guidelines across societies and disease specialties are a positive step, but concerted collaboration is required, with relevance to other long-term conditions

### Prevention gaps in type 2 diabetes

Given common risk factors across common diseases, integrated primary prevention approaches such as “ideal cardiovascular health” and the American Heart Association’s “Life’s Simple 7” are likely to have a greater impact than disease-specific approaches. Ideal cardiovascular health in individuals with CKD is associated with a lower risk of end-stage renal disease and mortality [19]. Our analyses in T2D show the importance of these factors and behaviours in the prevention of CVRD, to inform public health messages to patients.

### Treatment gaps in type 2 diabetes

Novel therapies such as SGLT-2 inhibitors have shown improved outcomes across organ systems in T2D [9], renewing interest in overlap between HF, CKD and T2D. However, we confirm practice gaps in utilisation, despite established and evidence-based therapies for CVD prevention in people with T2D. For example, only two-thirds and half of the T2D patients with atherosclerotic CVD were taking statins and aspirin, respectively. Improved drug therapy, smoking cessation and blood pressure and glycaemic control are crucial targets at individual and population levels to reduce CVRD in the context of T2D.

### Strengths and limitations

Although previous studies have considered lifetime risk of CVD, CKD and CVD in T2D [14–16, 28], this is the first study to date of lifetime risk of CVD and CKD together, and the first study across these diseases in T2D. We used lifetime risk, which gives a more complete picture of total disease burden, accounts for the effect of prolonged exposure to risk factors, remaining life expectancy and competing causes of death. Our analyses used nationally representative, population-based health record data, increasing the generalisability of our findings. There are some limitations. In the UK, our study population was 5% of the overall population. We did not have country-level data outside the UK and so our findings may need to be validated in other countries. Our analyses of risk use retrospective EHR data. In this study, we were unable to include asymptomatic CVRD or the incidence of CVRD without the need for hospitalisation in the outcome. The criteria for hospitalisation for CVRD may vary by clinician and institution and this was neither captured nor assessed in our analysis. We modelled using limited comorbidities, and absolute risk estimation methods do not allow for covariate adjustment throughout the lifecourse. For example, we did not study the impact of ethnicity. Residual confounding attributable to variables not covered by our data, such as diabetes duration, proteinuria and assessment of cardiac function might have influenced the results.

## Conclusions

**Fig. 4:**
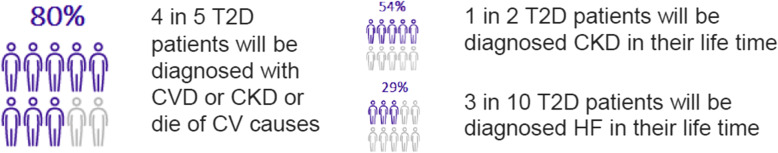
Summary figure. *Among T2D patients free of CVRD at >45 years of age

Four out of five individuals with T2D free from cardiovascular and renal disease, and nearly all those with a history of cardiovascular and renal disease, will develop major adverse renal and cardiovascular events over their lifetime. Early preventive measures in T2D patients are a clinical, public health and policy priority, including communicating using lifetime risk measures, integrated care across specialties, primary prevention strategies and improving the use of established therapies (Fig. 4).

## Supplementary Information

**Additional file 1: Table S1.** Baseline CVRD definitions. **Table S2.** Outcomes definitions. **Table S3.** Age-, sex- and baseline CVRD status-adjusted risk estimates and population attributable fractions (PAR) for non-modifiable and modifiable risk factors of HF in type 2 diabetes. **Table S4.** Age-, sex- and baseline CVRD status-adjusted risk estimates and population attributable fractions (PAR) for non-modifiable and modifiable risk factors of CKD in type 2 diabetes. **Table S5.** Age-, sex- and baseline CVRD status-adjusted risk estimates and population attributable fractions (PAR) for non-modifiable and modifiable risk factors of MI in type 2 diabetes. **Table S6.** Age-, sex- and baseline CVRD status-adjusted risk estimates and population attributable fractions (PAR) for non-modifiable and modifiable risk factors of Stroke in type 2 diabetes. **Table S7.** Age-, sex- and baseline CVRD status-adjusted risk estimates and population attributable fractions (PAR) for non-modifiable and modifiable risk factors of PAD in type 2 diabetes. **Table S8.** Age-, sex- and baseline CVRD status-adjusted risk estimates and population attributable fractions (PAR) for non-modifiable and modifiable risk factors of CVD death in type 2 diabetes. **Table S9.** Total event rates and follow up time in in type 2 diabetes by baseline CVRD status.

## Data Availability

The data that support the findings of this study are not available for sharing due to restrictions applicable to CPRD data: https://www.cprd.com/home/

## Abbreviations

CVRD: Cardiovascular and renal diseases
MARCE: Major adverse renal cardiovascular events
T2D: Type 2 diabetes
CVD: Cardiovascular diseases
EHR: Electronic health records
MI: Myocardial infarction
HF: Heart failure
TIA: Transient ischaemic attack
PAD: Peripheral artery disease
CKD: Chronic kidney disease
ARE: Attributable risk among the exposed
PAR: Population attributable risk
