# Correction to: Lifetime risk of cardiovascular-renal disease in type 2 diabetes: a population-based study in 473,399 individuals

**DOI:** 10.1186/s12916-022-02308-1

**Published:** 2022-03-23

**Authors:** Ruiqi Zhang, Jil Billy Mamza, Tamsin Morris, George Godfrey, Folkert W. Asselbergs, Spiros Denaxas, Harry Hemingway, Amitava Banerjee

**Affiliations:** 1 Robertson Centre for Biostatistics, Institute of Health and Wellbeing, University of Glasgow, Glasgow, UK; 2 Medical and Scientific Affairs, BioPharmaceuticals Medical, AstraZeneca, Cambridge, UK; 3 Institute of Health Informatics, University College London, London, UK; 4 Department of Cardiology, Division Heart & Lungs, University Medical Center Utrecht, Utrecht University, Utrecht, The Netherlands; 5 Institute of Cardiovascular Science, Faculty of Population Health Sciences, University College London, London, UK; 6 Health Data Research UK London, University College London, London, UK; 7 Barts Health NHS Trust, London, UK; 8 University College London Hospitals NHS Trust, London, UK

**BMC Medicine 20, 1–11 (2022)**

**https://doi.org/10.1186/s12916-022-02234-2**

The original article contained an error whereby the ‘HF’ sub-heading within Table 2 was mistakenly duplicated over the adjacent sub-headings. This has since been corrected.

