# Supplementary material for: Lifetime risk of cardiovascular-renal disease in type 2 diabetes: a population-based study in 473,399 individuals
Source: BMC Med. 2022 Feb 7;20:63. doi: 10.1186/s12916-022-02234-2 (PMC8822817; doi:10.1186/s12916-022-02234-2)
Supplement: Supplementary file 1 — Additional file 1: Table S1. Baseline CVRD definitions. Table S2. Outcomes definitions. Table S3. Age-, sex- and baseline CVRD status-adjusted risk estimates and population attributable fractions (PAR) for non-modifiable and modifiable risk factors of HF in type 2 diabetes. Table S4. Age-, sex- and baseline CVRD status-adjusted risk estimates and population attributable fractions (PAR) for non-modifiable and modifiable risk factors of CKD in type 2 diabetes. Table S5. Age-, sex- and baseline CVRD status-adjusted risk estimates and population attributable fractions (PAR) for non-modifiable and modifiable risk factors of MI in type 2 diabetes. Table S6. Age-, sex- and baseline CVRD status-adjusted risk estimates and population attributable fractions (PAR) for non-modifiable and modifiable risk factors of Stroke in type 2 diabetes. Table S7. Age-, sex- and baseline CVRD status-adjusted risk estimates and population attributable fractions (PAR) for non-modifiable and modifiable risk factors of PAD in type 2 diabetes. Table S8. Age-, sex- and baseline CVRD status-adjusted risk estimates and population attributable fractions (PAR) for non-modifiable and modifiable risk factors of CVD death in type 2 diabetes. Table S9. Total event rates and follow up time in in type 2 diabetes by baseline CVRD status. [file 12916_2022_2234_MOESM1_ESM.docx]

Additional File 1: Table S1. Baseline CVRD definitions

| Disease | Secondary hospitalization ICD 10 codes | Primary CPRD MedcodeIDs |
| --- | --- | --- |
| Myocardial infarction | I21-I22, I25.2, I25.6 | 810811000006116, 810821000006112, 451369010, 1738171000006114, 2173281000000112, 634751000006116, 256451015, 256452010, 256460011, 94884017, 219521000000119, 1786197015, 537751000006115, 37443015, 219531000000117, 1786198013, 350376014, 455641000006112, 299707016, 299708014, 455651000006114, 299709018, 457531000006110, 1234005010, 299710011, 299711010, 299712015, 116992017, 450322013, 1780501013, 299714019, 447324018, 1235655012, 967931000006114, 460681000006116, 1780491019, 299718016, 299719012, 1218860015, 1234306015, 299720018, 299721019, 109915012, 7844010, 494260016, 682481000006118, 2619484018, 1488382011, 299741012, 299742017, 1229885017, 459488019, 4031011, 4032016, 230021000006115, 32122016, 338974012, 458410010, 350354017, 2534674015, 299808017, 299811016, 299812011, 299813018, 118831000006118, 543291000006110, 813961000006116, 498031000006112, 67081000006119, 158601000006116, 158611000006118, 159001000006119, 100681000006116, 350379019, 208365015, 212061000006119, 212071000006114, 212091000006110, 455422014, 455423016, 216351000006118, 1778232017, 537191000006113, 2472090018, 350487017, 119261000006115, 453303015, 362461000006119, 300881018, 300882013 |
| Unstable angina | I20.0 | 854491000006113, 7844010, 7847015, 494260016, 72571000006115, 482811000006113, 7845011, 498328016, 299741012, 299742017, 299745015, 32122016, 1488382011, 931961000006117 |
| Angina pectoris | I20.1, I20.8, I20.9, I25.1, I25.5 + Nitrates | 251679016, 451370011, 264492012, 264493019, 264494013, 264495014, 264496010, 264497018, 451425016, 494261017, 299757012, 98087016, 59952018, 299763015, 482941000006119, 299758019, 36036010, 442204010, 338974012, 458410010, 350350014, 350348018, 299765010, 2537483013, 300874013 |
| **Heart failure** | I50, I11.0, I13.0, I13.2 | 251680018, 453099015, 1488591011, 1488804017, 833381000006119, 2159197017, 2159198010, 2117931000000116, 216184014, 1484918019, 2616470012, 2616471011, 2616472016, 2616473014, 404741000000119, 2122191000000110, 2645623019, 308011000000119, 451426015, 308301000000118, 1746171000000119, 2205951000000117, 1784061000006118, 2549208013, 1484917012, 2549697018, 308041000000118, 216246012, 2256811000000114, 303441000000116, 1705341000000110, 311561000000117, 1693911000000115, 1734081000000112, 2549243014, 2548316014, 303861000000118, 407181000000116, 303361000000111, 226181000000110, 2533628012, 2533629016, 2533630014, 2549089012, 407001000000113, 308231000000118, 308261000000111, 406801000000118, 407041000000111, 407061000000112, 407081000000115, 407101000000114, 138971000000111, 139061000000114, 139071000000119, 407441000000115, 1561941000006119, 1561951000006117, 72934016, 90135019, 728671000006119, 504901000006118, 741681000006111, 110659019, 741701000006114, 789941000006117, 82584011, 132655012, 1778488011, 350413012, 498953012, 139475013, 139482012, 70653017, 493287011, 206703015, 490972013, 510016018, 18472010, 147247018, 300179017, 300180019, 2675255018, 141306010, 1495417010, 411506018, 300190010, 94251011, 1647701000000118, 1661371000000112, 2227501000000110, 1816101000006113, 395772015, 139481017, 223981000000118, 300217019, 216207010, 1489358014, 2549128016, 316833010, 317955011, 350484012 |
| **CKD** | N17-N19, I12.0-I12.9, I13.1, I13.2, N08.3, E10.2, E11.2, E12.2, E13.2, E14.2, Z49, Z99.2 | 251923016, 251957012, 251956015, 354409011, 304031000000117, 304051000000112, 304071000000115, 304091000000116, 304111000000114, 557811000000119, 557831000000110, 595541000000119, 618391000000118, 595601000000118, 595661000000119, 618411000000118, 595721000000116, 618421000000112, 595811000000117, 618431000000114, 595871000000110, 618441000000117, 595931000000116, 618451000000119, 595991000000115, 618461000000116, 596051000000115, 618471000000111, 596131000000114, 618481000000113, 596191000000110, 618491000000110, 596261000000111, 618501000000116, 596321000000110, 618511000000119, 596401000000111, 618521000000113, 261136015, 2478488018, 2478492013, 2221251000000115, 384823013, 272134015, 394350012, 600481000006118, 349825015, 272139013, 272140010, 349856011, 360031000000113, 2675886019, 272141014, 272142019, 394352016, 743721000006115, 272153013, 637771000000113, 638111000000115, 501594013, 90471000006113, 272320011, 90461000006118, 478131000006115, 272325018, 372621000000114, 1549841000006116, 353974015, 272326017, 272327014, 394365013, 422992018, 272389014, 366671000000118, 386181000000111, 380101000000116, 1550021000006110, 380061000000118, 394639018, 619541000006116, 394640016, 106291000006118, 118218017, 278832011, 349998013, 2692168016, 357219012, 375841000000114, 278834012, 278835013, 228101000006113, 228131000006117, 773041000006111, 181021000006119, 1705621000000113, 228121000006115, 228211000006117, 278844014, 228231000006111, 228221000006113, 284213011, 408661000000117, 2160027017, 356557018, 454255014, 480871000006117, 299665018, 299673010, 2195121000000113, 2195661000000114, 2195721000000112, 1847111000006118, 207941012, 2210341000000113, 2238301000000119, 2237821000000118, 2238421000000115, 2240321000000114, 1870801000006111, 2240871000000116, 2240951000000115, 460091000006111, 1221119015, 303915016, 303916015, 509111010, 641471000006117, 2771041011, 215521000000116, 2767383018, 2767384012, 2773184015, 2767385013, 2767154014, 396711013, 1816581000006110, 177341000006111, 641491000006116, 354417015, 424791000006110, 424731000006111, 305191012, 305192017, 30043012, 714401000006112, 453312018, 1743241000006119, 2645965011, 350892013, 615611000006116, 324973015, 354619014, 413415016, 354610013, 2189151000000117, 2191221000000118, 1848741000006116, 2211671000000119, 441151000006115, 329115018, 329116017, 754061000006111, 177251000006119, 754781000006116, 176711000006111, 384641000006114, 460644012, 460680012, 460910019, 460911015, 1227722017, 460912010, 460913017, 1227723010, 461420012 |
| Atrial fibrillation | I48 | 2675253013, 636701000000115, 636721000000112, 350465014, 421235014, 256478018, 5669601000006117, 6016401000006115, 1823951000006111, 300132017, 3299911000006116, 82343012 |
| Stroke | I60-I66, G45 | 102489017, 104563015, 106392017, 106394016, 1130181000000113, 118689010, 1222398015, 122361000006113, 122371000006118, 122401000006115, 1227591017, 1227592012, 1230906015, 123441000006112, 123481000006118, 123491000006115, 123511000006114, 123521000006118, 124598014, 125470015, 126301000006113, 130375018, 145925010, 149551000006111, 149571000006118, 149701000006118, 158118014, 163261000006119, 1667741000000110, 1815931000006114, 1815951000006119, 1815971000006112, 1815981000006110, 200621000006117, 206411014, 2122001000000116, 216645017, 2171081000000116, 218511000000117, 2283831000000117, 2474651019, 2475877015, 2476091017, 2534198011, 25897016, 267311000006118, 294703019, 294711012, 297013012, 297233015, 297234014, 297238012, 297239016, 297786016, 297787013, 299342019, 299393011, 299394017, 299476010, 300242011, 300244012, 300253017, 300257016, 300276019, 300277011, 300287010, 300290016, 300294013, 300298011, 300303013, 300309012, 300310019, 300311015, 300312010, 300313017, 300314011, 300321011, 300322016, 300344014, 300345010, 300348012, 300349016, 300352012, 300353019, 300362017, 300363010, 300364016, 300366019, 300370010, 300371014, 300374018, 300379011, 300380014, 300395011, 300396012, 300398013, 300399017, 300401011, 300402016, 300403014, 300406018, 300407010, 300411016, 300418010, 300419019, 300935019, 300936018, 300939013, 300941014, 300942019, 300943012, 300956017, 302994014, 307879017, 307880019, 313568013, 313576010, 313577018, 316140013, 316519019, 316818019, 320735017, 320752018, 320771016, 320835014, 320836010, 345639010, 345650013, 345655015, 345658018, 345684012, 345771011, 345773014, 345780011, 359985011, 360778011, 370661000006114, 370701000006118, 370711000006115, 391042012, 391043019, 395777014, 395778016, 395780010, 395783012, 395788015, 402929011, 403081017, 405339016, 407291000006117, 411416011, 411453017, 411454011, 411518010, 413750011, 416991000006112, 428181000006115, 451123015, 451133011, 451134017, 451371010, 458505013, 458506014, 458507017, 480952017, 481028017, 483988011, 495394013, 497021000006113, 499739014, 502878012, 503469016, 503791000006114, 524511000006116, 524541000006117, 542251000006112, 542261000006114, 542831000006116, 543071000006115, 543141000006110, 543251000006116, 57341000006119, 58046010, 605461000006117, 605471000006112, 605481000006110, 605491000006113, 605501000006117, 622321000000113, 64469014, 67501000006115, 67511000006117, 744901000006114, 744921000006116, 746571000006116, 748941000006115, 761411000006111, 769281000006119, 800691000006113, 809421000006116, 928621000006111, 95931000006111 |
| Hemorrhagic | I60-I62 |  |
| Ischemic | I63 |  |
| Transitory ischemic attack | G45 |  |
| Peripheral artery disease | I70.2, I73.9, I74.2-9 | 450665015, 262274019, 639341000006115, 530051000006117, 501011000006118, 271416011, 271431019, 639371000006111, 530061000006115, 224121000000118, 410961010, 530071000006110, 488631000006116, 410962015, 271444012, 271445013, 271509013, 394323019, 639471000006115, 530201000006110, 639491000006119, 640081000006119, 530191000006112, 668731000006114, 639391000006112, 639521000006117, 530091000006111, 530221000006117, 530231000006119, 785611000006113, 271867017, 271868010, 394326010, 349652010, 641691000006118, 410964019, 394328011, 394329015, 410965018, 271880019, 271881015, 394330013, 410966017, 394331012, 410967014, 394332017, 411214011, 455900010, 271900018, 271901019, 271905011, 455901014, 271907015, 271908013, 394338018, 32181000006113, 349670013, 349669012, 349668016, 349667014, 639431000006118, 639581000006118, 639421000006116, 639551000006114, 639451000006113, 639441000006111, 639561000006111, 639541000006112, 639461000006110, 639601000006111, 31361000006114, 31351000006112, 394339014, 349666017, 349665018, 349663013, 349662015, 349661010, 530131000006113, 530811000006112, 530141000006115, 530821000006116, 530151000006118, 530831000006118, 530161000006116, 530841000006111, 530111000006119, 530121000006110, 530781000006110, 530171000006111, 530791000006113, 668741000006116, 36061000006118, 272003016, 394340011, 410976019, 410977011, 410974016, 411078012, 641681000006116, 641701000006118, 272013012, 272014018, 201771000006117, 201761000006112, 272017013, 272018015, 272019011, 272020017, 187331000006112, 272022013, 394341010, 410981011, 410979014, 410978018, 272029016, 272030014, 272031013, 272032018, 272033011, 272035016, 272034017, 272041011, 272042016, 24571000006113, 272052017, 239491000006117, 272055015, 272056019, 272059014, 272061017, 238621000006112, 1548341000006118, 272074011, 272075012, 272076013, 272077016, 272078014, 286570019, 616441000006113, 616591000006117, 616581000006115, 787101000006114, 674961000006118, 292523015, 772141000006118, 280561000006111, 84961000006116, 84591000006119, 913931000006116, 459308015, 223991000000116, 300433019, 300434013, 395791015, 350535018, 742481000006118, 350533013, 87208017, 87210015, 300503012, 300504018, 300505017, 300506016, 443199013, 300510018, 302011, 300511019, 41903014, 454901000006118, 454891000006117, 503681010, 300512014, 124092018, 61977017, 300514010, 235911000006116, 105536013, 411512011, 300515011, 350564010, 300539018, 300540016, 300541017, 300542012, 300547018, 300550015, 300553018, 300554012, 300555013, 300556014, 300963017, 357895013, 309177010, 357894012, 357893018, 317347010, 317348017, 317351012, 1222508013 |

Additional File 1: Table S2. Outcomes definitions

| Variable | ICD-10 Definition |
| --- | --- |
| All-cause death | Death of any cause |
| Cardiovascular death | Death with any “I” diagnosis as underlying cause of death |
| Myocardial infarction | I21-I22, I25.2, I25.6 |
| Stroke | I60-I63 |
| Heart failure | I60-I66, G45 |
| CKD | N17-N19, I12.0-I2.9, I13.1, I13.2, N08.3, E10.2, E11.2, E12.2, E13.2, E14.2, Z49, Z99.2 |
| PAD | I70.2, I73.9, I74.2-9 |

Additional File 1: Table S3. Age-, sex- and baseline CVRD status-adjusted risk estimates and population attributable fractions (PAR) for non-modifiable and modifiable risk factors of HF in type 2 diabetes

| Risk Factors | Category | OR (95%CI) | Estimated Risk | ARE | % of events (Pe) | PAR |  |
| --- | --- | --- | --- | --- | --- | --- | --- |
| Non-modifiable |  |  |  |  |  |  |  |
| Age | <45 | 1 | 0.009 |  | 11.1 |  |  |
|  | 45-54 | 1.67 (1.54, 1.80) | 0.015 | 0.397 | 17.5 | 0.069 |  |
|  | 55-64 | 2.90 (2.69, 3.11) | 0.025 | 0.649 | 23.4 | 0.152 |  |
|  | 65-74 | 5.20 (4.85, 5.59) | 0.044 | 0.801 | 24.3 | 0.195 |  |
|  | 75-84 | 7.85 (7.32, 8.43) | 0.065 | 0.865 | 17.8 | 0.154 |  |
|  | 85+ | 8.23 (7.64, 8.88) | 0.068 | 0.871 | 5.9 | 0.052 |  |
| Sex | Female | 1 | 0.163 |  | 45.7 |  |  |
|  | Male | 1.11 (1.08, 1.14) | 0.178 | 0.085 | 54.3 | 0.046 |  |
| Modifiable |  |  |  |  |  |  |  |
| Comorbidities | CVRD free | 1 | 0.017 |  | 58.5 |  |  |
|  | HF only | 9.65 (8.84, 10.54) | 0.140 | 0.882 | 1.1 | 0.010 |  |
|  | CKD only | 1.64 (1.56, 1.73) | 0.027 | 0.386 | 7.4 | 0.029 |  |
|  | MI only | 2.27 (1.94, 2.66) | 0.037 | 0.550 | 0.6 | 0.003 |  |
|  | Stroke only | 1.47 (1.37, 1.59) | 0.024 | 0.317 | 3.6 | 0.011 |  |
|  | PAD only | 2.10 (1.91, 2.32) | 0.034 | 0.515 | 1.2 | 0.006 |  |
|  | Others | 4.03 (3.90, 4.16) | 0.064 | 0.739 | 27.6 | 0.204 |  |
| BMI | Normal (20-24) | 1 | 0.031 |  | 12.7 |  |  |
|  | Under weight (<20) | 0.75 (0.67, 0.84) | 0.023 | -0.320 | 1.6 | -0.005 |  |
|  | Over weight (25-29) | 1.19 (1.14, 1.24) | 0.036 | 0.154 | 31.6 | 0.049 |  |
|  | Obese (30-39) | 1.63 (1.56, 1.69) | 0.049 | 0.373 | 43.3 | 0.162 |  |
|  | Severely obese (40+) | 2.59 (2.44, 2.74) | 0.076 | 0.595 | 10.8 | 0.064 |  |
| Smoking | Never | 1 | 0.046 |  | 19.7 |  |  |
|  | Former | 1.03 (0.99, 1.06) | 0.047 | 0.024 | 46.3 | 0.011 |  |
|  | Current | 1.00 (0.97, 1.04) | 0.046 | 0.002 | 34.0 | 0.001 |  |
| Hba1c | < 7% - good control | 1 | 0.037 |  | 50.8 |  |  |
|  | 7-10% - borderline | 1.35 (1.31, 1.39) | 0.049 | 0.249 | 40.9 | 0.102 |  |
|  | 10% - bad control | 1.83 (1.73, 1.92) | 0.066 | 0.435 | 8.3 | 0.036 |  |
| Hypertension | SBP<150 and DBP<100 | 1 | 0.046 |  | 83.3 |  |  |
|  | SBP>150 or DBP>100 | 1.19 (1.16, 1.23) | 0.054 | 0.155 | 16.7 | 0.026 |  |
| High cholesterol | HDL≥1 & LDL≤3 & Triglyceride ≤2.3 | 1 | 0.032 |  | 38.4 |  | |
|  | HDL≤1 or LDL≥3 or Triglyceride ≥2.3 | 1.10 (1.07, 1.13) | 0.035 | 0.086 | 61.6 | 0.053 | |
| Physical activity | Inactive | 2.04 (1.87, 2.22) | 0.036 | 0.500 | 43.0 | 0.215 | |
|  | Moderately inactive | 1.13 (1.01, 1.27) | 0.020 | 0.113 | 9.1 | 0.010 | |
|  | Moderately active | 1.33 (1.22, 1.45) | 0.024 | 0.243 | 36.0 | 0.087 | |
|  | Active | 1 | 0.018 |  | 12.0 |  | |
| Overall cardiovascular health* | Ideal | 1 | 0.019 |  | 1.0 |  | |
|  | ≥1 risk factor | 1.81 (1.65, 2.00) | 0.034 | 0.440 | 99.0 | 0.435 | |

*Risk factors: BMI ≥30, current smoker, HbA1C≥7%, hypertension, high cholesterol, inactive

Additional File 1: Table S4. Age-, sex- and baseline CVRD status-adjusted risk estimates and population attributable fractions (PAR) for non-modifiable and modifiable risk factors of CKD in type 2 diabetes

| Risk Factors | | Category | OR (95%CI) | Estimated Risk | ARE | % of events  (Pe) | PAR |
| --- | --- | --- | --- | --- | --- | --- | --- |
| Non-modifiable | |  |  |  |  |  |  |
| Age | | <45 | 1 | 0.012 |  | 11.1 |  |
|  | | 45-54 | 1.41 (1.34, 1.48) | 0.017 | 0.286 | 17.5 | 0.050 |
|  | | 55-64 | 2.40 (2.29, 2.52) | 0.028 | 0.576 | 23.4 | 0.135 |
|  | | 65-74 | 4.47 (4.27, 4.68) | 0.052 | 0.767 | 24.3 | 0.186 |
|  | | 75-84 | 6.97 (6.66, 7.30) | 0.078 | 0.846 | 17.8 | 0.151 |
|  | | 85+ | 6.72 (6.38, 7.07) | 0.076 | 0.841 | 5.9 | 0.050 |
| Sex | | Female | 1 | 0.273 |  | 45.7 |  |
|  | | Male | 1.06 (1.04, 1.08) | 0.285 | 0.042 | 54.3 | 0.023 |
| Modifiable | |  |  |  |  |  |  |
| Comorbidities | | CVRD free | 1 | 0.025 |  | 58.5 |  |
|  | | HF only | 2.31 (2.11, 2.53) | 0.055 | 0.553 | 1.1 | 0.006 |
|  | | CKD only | 4.08 (3.94, 4.23) | 0.093 | 0.737 | 7.4 | 0.054 |
|  | | MI only | 1.37 (1.19, 1.58) | 0.033 | 0.264 | 0.6 | 0.002 |
|  | | Stroke only | 1.48 (1.39, 1.56) | 0.036 | 0.314 | 3.6 | 0.011 |
|  | | PAD only | 1.97 (1.82, 2.13) | 0.047 | 0.480 | 1.2 | 0.006 |
|  | | Others | 2.93 (2.86, 3.01) | 0.069 | 0.643 | 27.6 | 0.177 |
| BMI | | Normal (20-24) | 1 | 0.042 |  | 12.7 |  |
|  | | Under weight (<20) | 0.74 (0.68, 0.81) | 0.032 | -0.330 | 1.6 | -0.005 |
|  | | Over weight (25-29) | 1.14 (1.11, 1.18) | 0.048 | 0.120 | 31.6 | 0.038 |
|  | | Obese (30-39) | 1.37 (1.32, 1.42) | 0.057 | 0.258 | 43.3 | 0.112 |
|  | | Severely obese (40+) | 1.88 (1.79, 1.97) | 0.076 | 0.448 | 10.8 | 0.048 |
| Smoking | | Never | 1 | 0.054 |  | 19.7 |  |
|  | | Former | 1.03 (1.00, 1.06) | 0.055 | 0.030 | 46.3 | 0.014 |
|  | | Current | 1.02 (0.99, 1.05) | 0.055 | 0.021 | 34.0 | 0.007 |
| Hba1c | | < 7% - good control | 1 | 0.043 |  | 50.8 |  |
|  | | 7-10% - borderline | 1.39 (1.35, 1.42) | 0.059 | 0.266 | 40.9 | 0.109 |
|  | | 10% - bad control | 1.86 (1.78, 1.94) | 0.077 | 0.443 | 8.3 | 0.037 |
| Hypertension | | SBP<150 and DBP<100 | 1 | 0.053 |  | 83.3 |  |
|  | | SBP>150 or DBP>100 | 1.24 (1.20, 1.27) | 0.065 | 0.182 | 16.7 | 0.031 |
| High cholesterol | HDL≥1 & LDL≤3 & Triglyceride ≤2.3 | | 1 | 0.052 |  | 38.4 |  |
|  | HDL≤1 or LDL≥3 or Triglyceride ≥2.3 | | 1.09 (1.07, 1.12) | 0.056 | 0.079 | 61.6 | 0.048 |
| Physical activity | Inactive | | 1.96 (1.83, 2.09) | 0.057 | 0.474 | 43.0 | 0.204 |
|  | Moderately inactive | | 1.08 (0.98, 1.18) | 0.032 | 0.069 | 9.1 | 0.006 |
|  | Moderately active | | 1.35 (1.26, 1.44) | 0.040 | 0.251 | 36.0 | 0.090 |
|  | Active | | 1 | 0.030 |  | 12.0 |  |
| Overall cardiovascular health* | Ideal | | 1 | 0.033 |  | 1.0 |  |
|  | ≥1 risk factor | | 1.74 (1.61, 1.88) | 0.055 | 0.412 | 99.0 | 0.408 |

*Risk factors: BMI ≥30, current smoker, HbA1C≥7%, hypertension, high cholesterol, inactive

Additional File 1: Table S5. Age-, sex- and baseline CVRD status-adjusted risk estimates and population attributable fractions (PAR) for non-modifiable and modifiable risk factors of MI in type 2 diabetes

| Risk Factors | | Category | OR (95%CI) | Estimated Risk | ARE | % of events (Pe) | PAR |
| --- | --- | --- | --- | --- | --- | --- | --- |
| Non-modifiable | |  |  |  |  |  |  |
| Age | | <45 | 1 | 0.012 |  | 11.1 |  |
|  | | 45-54 | 1.52 (1.42, 1.62) | 0.018 | 0.337 | 17.5 | 0.059 |
|  | | 55-64 | 1.89 (1.78, 2.01) | 0.022 | 0.467 | 23.4 | 0.109 |
|  | | 65-74 | 2.26 (2.13, 2.40) | 0.026 | 0.552 | 24.3 | 0.134 |
|  | | 75-84 | 2.34 (2.21, 2.49) | 0.027 | 0.567 | 17.8 | 0.101 |
|  | | 85+ | 1.79 (1.68, 1.92) | 0.021 | 0.437 | 5.9 | 0.026 |
| Sex | | Female | 1 | 0.095 |  | 45.7 |  |
|  | | Male | 1.50 (1.46, 1.55) | 0.136 | 0.303 | 54.3 | 0.165 |
| Modifiable | |  |  |  |  |  |  |
| Comorbidities | | CVRD free | 1 | 0.009 |  | 58.5 |  |
|  | | HF only | 2.03 (1.75, 2.35) | 0.018 | 0.502 | 1.1 | 0.006 |
|  | | CKD only | 1.66 (1.56, 1.78) | 0.015 | 0.395 | 7.4 | 0.029 |
|  | | MI only | 16.89 (15.01, 19.02) | 0.131 | 0.933 | 0.6 | 0.005 |
|  | | Stroke only | 1.81 (1.65, 1.98) | 0.016 | 0.444 | 3.6 | 0.016 |
|  | | PAD only | 2.58 (2.31, 2.89) | 0.022 | 0.607 | 1.2 | 0.008 |
|  | | Others | 9.49 (9.15, 9.84) | 0.078 | 0.887 | 27.6 | 0.244 |
| BMI | | Normal (20-24) | 1 | 0.024 |  | 12.7 |  |
|  | | Under weight (<20) | 0.71 (0.63, 0.81) | 0.017 | -0.391 | 1.6 | -0.006 |
|  | | Over weight (25-29) | 1.10 (1.05, 1.14) | 0.027 | 0.085 | 31.6 | 0.027 |
|  | | Obese (30-39) | 1.11 (1.06, 1.16) | 0.027 | 0.096 | 43.3 | 0.042 |
|  | | Severely obese (40+) | 1.00 (0.93, 1.06) | 0.024 | -0.005 | 10.8 | -0.001 |
| Smoking | | Never | 1 | 0.024 |  | 19.7 |  |
|  | | Former | 1.09 (1.05, 1.13) | 0.026 | 0.081 | 46.3 | 0.037 |
|  | | Current | 1.14 (1.10, 1.19) | 0.027 | 0.122 | 34.0 | 0.042 |
| Hba1c | | < 7% - good control | 1 | 0.021 |  | 50.8 |  |
|  | | 7-10% - borderline | 1.28 (1.25, 1.32) | 0.027 | 0.216 | 40.9 | 0.088 |
|  | | 10% - bad control | 1.48 (1.40, 1.56) | 0.031 | 0.316 | 8.3 | 0.026 |
| Hypertension | | SBP<150 and DBP<100 | 1 | 0.025 |  | 83.3 |  |
|  | | SBP>150 or DBP>100 | 1.11 (1.07, 1.15) | 0.028 | 0.096 | 16.7 | 0.016 |
| High cholesterol | HDL≥1 & LDL≤3 & Triglyceride ≤2.3 | | 1 | 0.017 |  | 38.4 |  |
|  | HDL≤1 or LDL≥3 or Triglyceride ≥2.3 | | 1.17 (1.13, 1.20) | 0.020 | 0.139 | 61.6 | 0.086 |
| Physical activity | Inactive | | 1.42 (1.31, 1.54) | 0.019 | 0.294 | 43.0 | 0.126 |
|  | Moderately inactive | | 1.01 (0.90, 1.13) | 0.013 | 0.009 | 9.1 | 0.001 |
|  | Moderately active | | 1.35 (1.24, 1.46) | 0.018 | 0.255 | 36.0 | 0.092 |
|  | Active | | 1 | 0.013 |  | 12.0 |  |
| Overall cardiovascular health* | Ideal | | 1 | 0.022 |  | 1.0 |  |
|  | ≥1 risk factor | | 1.19 (1.09, 1.31) | 0.026 | 0.159 | 99.0 | 0.157 |

*Risk factors: BMI ≥30, current smoker, HbA1C≥7%, hypertension, high cholesterol, inactive

Additional File 1: Table S6. Age-, sex- and baseline CVRD status-adjusted risk estimates and population attributable fractions (PAR) for non-modifiable and modifiable risk factors of Stroke in type 2 diabetes

| Risk Factors | | Category | | OR (95%CI) | Estimated Risk | ARE | % of events (Pe) | PAR |
| --- | --- | --- | --- | --- | --- | --- | --- | --- |
| Non-modifiable | |  | |  |  |  |  |  |
| Age | | <45 | | 1 | 0.003 |  | 11.1 |  |
|  | | 45-54 | | 1.87 (1.70, 2.05) | 0.006 | 0.463 | 17.5 | 0.081 |
|  | | 55-64 | | 2.77 (2.53, 3.03) | 0.009 | 0.637 | 23.4 | 0.149 |
|  | | 65-74 | | 4.88 (4.47, 5.33) | 0.016 | 0.792 | 24.3 | 0.193 |
|  | | 75-84 | | 6.91 (6.33, 7.55) | 0.022 | 0.852 | 17.8 | 0.152 |
|  | | 85+ | | 6.52 (5.94, 7.17) | 0.021 | 0.844 | 5.9 | 0.050 |
| Sex | | Female | | 1 | 0.072 |  | 45.7 |  |
|  | | Male | | 1.03 (1.00, 1.07) | 0.075 | 0.031 | 54.3 | 0.017 |
| Modifiable | |  | |  |  |  |  |  |
| Comorbidities | | CVRD free | | 1 | 0.010 |  | 58.5 |  |
|  | | HF only | | 1.55 (1.33, 1.80) | 0.015 | 0.351 | 1.1 | 0.004 |
|  | | CKD only | | 1.33 (1.25, 1.42) | 0.013 | 0.246 | 7.4 | 0.018 |
|  | | MI only | | 1.80 (1.47, 2.21) | 0.017 | 0.440 | 0.6 | 0.003 |
|  | | Stroke only | | 3.13 (2.91, 3.37) | 0.029 | 0.674 | 3.6 | 0.024 |
|  | | PAD only | | 2.22 (1.98, 2.49) | 0.021 | 0.545 | 1.2 | 0.007 |
|  | | Others | | 2.17 (2.08, 2.26) | 0.020 | 0.534 | 27.6 | 0.147 |
| BMI | | Normal (20-24) | | 1 | 0.017 |  | 12.7 |  |
|  | | Under weight (<20) | | 0.79 (0.69, 0.90) | 0.013 | -0.262 | 1.6 | -0.004 |
|  | | Over weight (25-29) | | 1.04 (0.99, 1.09) | 0.017 | 0.035 | 31.6 | 0.011 |
|  | | Obese (30-39) | | 0.98 (0.93, 1.03) | 0.016 | -0.024 | 43.3 | -0.010 |
|  | | Severely obese (40+) | | 0.87 (0.80, 0.94) | 0.015 | -0.146 | 10.8 | -0.016 |
| Smoking | | Never | | 1 | 0.017 |  | 19.7 |  |
|  | | Former | | 0.91 (0.87, 0.95) | 0.015 | -0.100 | 46.3 | -0.046 |
|  | | Current | | 1.04 (1.00, 1.09) | 0.018 | 0.041 | 34.0 | 0.014 |
| Hba1c | | < 7% - good control | | 1 | 0.013 |  | 50.8 |  |
|  | | 7-10% - borderline | | 1.30 (1.26, 1.35) | 0.017 | 0.228 | 40.9 | 0.093 |
|  | | 10% - bad control | | 1.63 (1.53, 1.74) | 0.022 | 0.382 | 8.3 | 0.032 |
| Hypertension | | SBP<150 and DBP<100 | | 1 | 0.016 |  | 83.3 |  |
|  | | SBP>150 or DBP>100 | | 1.31 (1.25, 1.36) | 0.021 | 0.231 | 16.7 | 0.039 |
| High cholesterol | HDL≥1 & LDL≤3 & Triglyceride ≤2.3 | | 1 | | 0.015 |  | 38.4 |  |
|  | HDL≤1 or LDL≥3 or Triglyceride ≥2.3 | | 1.08 (1.04, 1.12) | | 0.017 | 0.069 | 61.6 | 0.042 |
| Physical activity | Inactive | | 1.61 (1.44, 1.81) | | 0.015 | 0.377 | 43.0 | 0.162 |
|  | Moderately inactive | | 1.05 (0.90, 1.23) | | 0.010 | 0.047 | 9.1 | 0.004 |
|  | Moderately active | | 1.47 (1.31, 1.65) | | 0.014 | 0.318 | 36.0 | 0.114 |
|  | Active | | 1 | | 0.010 |  | 12.0 |  |
| Overall cardiovascular health* | Ideal | | 1 | | 0.012 |  | 1.0 |  |
|  | ≥1 risk factor | | 1.32 (1.17, 1.48) | | 0.016 | 0.237 | 99.0 | 0.235 |

*Risk factors: BMI ≥30, current smoker, HbA1C≥7%, hypertension, high cholesterol, inactive

Additional File 1: Table S7. Age-, sex- and baseline CVRD status-adjusted risk estimates and population attributable fractions (PAR) for non-modifiable and modifiable risk factors of PAD in type 2 diabetes

| Risk Factors | | Category | | OR (95%CI) | Estimated Risk | ARE | % of events (Pe) | PAR |
| --- | --- | --- | --- | --- | --- | --- | --- | --- |
| Non-modifiable | |  | |  |  |  |  |  |
| Age | | <45 | | 1 | 0.003 |  | 11.1 |  |
|  | | 45-54 | | 1.77 (1.59, 1.97) | 0.006 | 0.435 | 17.5 | 0.076 |
|  | | 55-64 | | 2.52 (2.28, 2.79) | 0.008 | 0.602 | 23.4 | 0.141 |
|  | | 65-74 | | 3.16 (2.86, 3.49) | 0.010 | 0.681 | 24.3 | 0.166 |
|  | | 75-84 | | 3.17 (2.87, 3.51) | 0.010 | 0.682 | 17.8 | 0.121 |
|  | | 85+ | | 2.34 (2.09, 2.62) | 0.008 | 0.571 | 5.9 | 0.034 |
| Sex | | Female | | 1 | 0.048 |  | 45.7 |  |
|  | | Male | | 1.53 (1.47, 1.60) | 0.072 | 0.331 | 54.3 | 0.180 |
| Modifiable | |  | |  |  |  |  |  |
| Comorbidities | | CVRD free | | 1 | 0.004 |  | 58.5 |  |
|  | | HF only | | 1.89 (1.55, 2.32) | 0.008 | 0.470 | 1.1 | 0.005 |
|  | | CKD only | | 1.67 (1.53, 1.83) | 0.007 | 0.401 | 7.4 | 0.030 |
|  | | MI only | | 2.05 (1.58, 2.66) | 0.009 | 0.510 | 0.6 | 0.003 |
|  | | Stroke only | | 1.92 (1.70, 2.16) | 0.008 | 0.477 | 3.6 | 0.017 |
|  | | PAD only | | 21.05 (19.37, 22.87) | 0.084 | 0.948 | 1.2 | 0.012 |
|  | | Others | | 5.19 (4.94, 5.46) | 0.022 | 0.804 | 27.6 | 0.222 |
| BMI | | Normal (20-24) | | 1 | 0.013 |  | 12.7 |  |
|  | | Under weight (<20) | | 0.97 (0.83, 1.13) | 0.012 | -0.035 | 1.6 | -0.001 |
|  | | Over weight (25-29) | | 0.96 (0.90, 1.01) | 0.012 | -0.043 | 31.6 | -0.014 |
|  | | Obese (30-39) | | 0.96 (0.91, 1.02) | 0.012 | -0.041 | 43.3 | -0.018 |
|  | | Severely obese (40+) | | 0.86 (0.79, 0.94) | 0.011 | -0.160 | 10.8 | -0.017 |
| Smoking | | Never | | 1 | 0.009 |  | 19.7 |  |
|  | | Former | | 1.26 (1.19, 1.33) | 0.011 | 0.204 | 46.3 | 0.094 |
|  | | Current | | 1.59 (1.50, 1.68) | 0.014 | 0.368 | 34.0 | 0.125 |
| Hba1c | | < 7% - good control | | 1 | 0.008 |  | 50.8 |  |
|  | | 7-10% - borderline | | 1.49 (1.43, 1.55) | 0.012 | 0.324 | 40.9 | 0.133 |
|  | | 10% - bad control | | 2.27 (2.13, 2.43) | 0.018 | 0.555 | 8.3 | 0.046 |
| Hypertension | | SBP<150 and DBP<100 | | 1 | 0.011 |  | 83.3 |  |
|  | | SBP>150 or DBP>100 | | 1.32 (1.26, 1.38) | 0.015 | 0.239 | 16.7 | 0.040 |
| High cholesterol | HDL≥1 & LDL≤3 & Triglyceride ≤2.3 | | 1 | | 0.010 |  | 38.4 |  |
|  | HDL≤1 or LDL≥3 or Triglyceride ≥2.3 | | 1.22 (1.17, 1.27) | | 0.012 | 0.180 | 61.6 | 0.111 |
| Physical activity | Inactive | | 2.20 (1.93, 2.50) | | 0.014 | 0.542 | 43.0 | 0.233 |
|  | Moderately inactive | | 1.24 (1.04, 1.49) | | 0.008 | 0.194 | 9.1 | 0.018 |
|  | Moderately active | | 1.55 (1.35, 1.76) | | 0.010 | 0.351 | 36.0 | 0.126 |
|  | Active | | 1 | | 0.006 |  | 12.0 |  |
| Overall cardiovascular health* | Ideal | | 1 | | 0.008 |  | 1.0 |  |
|  | ≥1 risk factor | | 1.55 (1.34, 1.78) | | 0.012 | 0.351 | 99.0 | 0.347 |

*Risk factors: BMI ≥30, current smoker, HbA1C≥7%, hypertension, high cholesterol, inactive

Additional File 1: Table S8. Age-, sex- and baseline CVRD status-adjusted risk estimates and population attributable fractions (PAR) for non-modifiable and modifiable risk factors of CVD death in type 2 diabetes

| Risk Factors | | Category | | OR (95%CI) | Estimated Risk | ARE | % of events (Pe) | PAR |
| --- | --- | --- | --- | --- | --- | --- | --- | --- |
| Non-modifiable | |  | |  |  |  |  |  |
| Age | | <45 | | 1 | 0.004 |  | 11.1 |  |
|  | | 45-54 | | 1.97 (1.79, 2.17) | 0.007 | 0.490 | 17.5 | 0.086 |
|  | | 55-64 | | 3.88 (3.55, 4.24) | 0.014 | 0.739 | 23.4 | 0.173 |
|  | | 65-74 | | 8.57 (7.85, 9.34) | 0.030 | 0.880 | 24.3 | 0.214 |
|  | | 75-84 | | 18.36 (16.83, 20.03) | 0.063 | 0.942 | 17.8 | 0.168 |
|  | | 85+ | | 29.33 (26.82, 32.07) | 0.097 | 0.962 | 5.9 | 0.057 |
| Sex | | Female | | 1 | 0.145 |  | 45.7 |  |
|  | | Male | | 1.22 (1.18, 1.25) | 0.171 | 0.151 | 54.3 | 0.082 |
| Modifiable | |  | |  |  |  |  |  |
| Comorbidities | | CVRD free | | 1 | 0.016 |  | 58.5 |  |
|  | | HF only | | 3.47 (3.15, 3.82) | 0.052 | 0.700 | 1.1 | 0.008 |
|  | | CKD only | | 1.61 (1.53, 1.68) | 0.025 | 0.371 | 7.4 | 0.027 |
|  | | MI only | | 2.45 (2.12, 2.84) | 0.037 | 0.583 | 0.6 | 0.003 |
|  | | Stroke only | | 2.37 (2.22, 2.52) | 0.036 | 0.569 | 3.6 | 0.020 |
|  | | PAD only | | 2.77 (2.54, 3.02) | 0.042 | 0.629 | 1.2 | 0.008 |
|  | | Others | | 3.29 (3.19, 3.39) | 0.049 | 0.685 | 27.6 | 0.189 |
| BMI | | Normal (20-24) | | 1 | 0.037 |  | 12.7 |  |
|  | | Under weight (<20) | | 1.16 (1.06, 1.27) | 0.043 | 0.133 | 1.6 | 0.002 |
|  | | Over weight (25-29) | | 0.90 (0.87, 0.94) | 0.034 | -0.104 | 31.6 | -0.033 |
|  | | Obese (30-39) | | 0.96 (0.92, 0.99) | 0.036 | -0.042 | 43.3 | -0.018 |
|  | | Severely obese (40+) | | 1.40 (1.32, 1.48) | 0.051 | 0.275 | 10.8 | 0.030 |
| Smoking | | Never | | 1 | 0.037 |  | 19.7 |  |
|  | | Former | | 0.96 (0.93, 0.99) | 0.035 | -0.038 | 46.3 | -0.018 |
|  | | Current | | 1.11 (1.07, 1.15) | 0.040 | 0.092 | 34.0 | 0.031 |
| Hba1c | | < 7% - good control | | 1 | 0.029 |  | 50.8 |  |
|  | | 7-10% - borderline | | 1.35 (1.32, 1.39) | 0.039 | 0.252 | 40.9 | 0.103 |
|  | | 10% - bad control | | 2.00 (1.90, 2.11) | 0.057 | 0.485 | 8.3 | 0.041 |
| Hypertension | | SBP<150 and DBP<100 | | 1 | 0.037 |  | 83.3 |  |
|  | | SBP>150 or DBP>100 | | 1.24 (1.20, 1.28) | 0.045 | 0.187 | 16.7 | 0.031 |
| High cholesterol | HDL≥1 & LDL≤3 & Triglyceride ≤2.3 | | 1 | | 0.033 |  | 38.4 |  |
|  | HDL≤1 or LDL≥3 or Triglyceride ≥2.3 | | 1.13 (1.10, 1.16) | | 0.038 | 0.111 | 61.6 | 0.069 |
| Physical activity | Inactive | | 2.41 (2.21, 2.63) | | 0.037 | 0.576 | 43.0 | 0.248 |
|  | Moderately inactive | | 1.13 (1.00, 1.27) | | 0.018 | 0.111 | 9.1 | 0.010 |
|  | Moderately active | | 1.50 (1.38, 1.64) | | 0.023 | 0.330 | 36.0 | 0.119 |
|  | Active | | 1 | | 0.016 |  | 12.0 |  |
| Overall cardiovascular health* | Ideal | | 1 | | 0.020 |  | 1.0 |  |
|  | ≥1 risk factor | | 1.91 (1.75, 2.10) | | 0.037 | 0.468 | 99.0 | 0.463 |

*Risk factors: BMI ≥30, current smoker, HbA1C≥7%, hypertension, high cholesterol, inactive

|  |  | **All T2D** | **CVRD free** | **HF only** | **CKD only** | **MI only** | **Stroke only** | **PAD only** | **Multiple CVRD** |
| --- | --- | --- | --- | --- | --- | --- | --- | --- | --- |
|  | N | 473399 | 276941 | 5310 | 35019 | 2721 | 17044 | 5898 | 126615 |
| All-cause death | FU (patient-years) | 2582109 | 1553831 | 24552 | 190430 | 15361 | 88417 | 32866 | 662136 |
|  | n (n/N) | 102748 (21.7%) | 31070 (11.2%) | 2132 (40.2%) | 11276 (32.2%) | 810 (29.8%) | 5615 (32.9%) | 2152 (36.5%) | 47468 (37.5%) |
| CV death | FU (patient-years) | 2582109 | 1553831 | 24552 | 190430 | 15361 | 88417 | 32866 | 662136 |
|  | n (n/N) | 57797 (12.2%) | 12961 (4.7%) | 1359 (25.6%) | 5378 (15.4%) | 476 (17.5%) | 3214 (18.9%) | 1139 (19.3%) | 31818 (25.1%) |
| MI | FU (patient-years) | 2419201 | 1504783 | 23359 | 183625 | 12094 | 84840 | 31277 | 565408 |
|  | n (n/N) | 50152 (10.6%) | 8355 (3.0%) | 392 (7.4%) | 2016 (5.8%) | 1154 (42.4%) | 1075 (6.3%) | 568 (9.6%) | 36243 (28.6%) |
| Stroke | FU (patient-years) | 2475371 | 1503435 | 23590 | 182993 | 14623 | 81152 | 31144 | 624632 |
|  | n (n/N) | 26528 (5.6%) | 8200 (3.0%) | 357 (6.7%) | 2218 (6.3%) | 212 (7.8%) | 2216 (13.0%) | 545 (9.2%) | 12473 (9.9%) |
| Pad | FU (patient-years) | 2486108 | 1513646 | 23773 | 185342 | 14909 | 85686 | 26559 | 622125 |
|  | n (n/N) | 19894 (4.2%) | 3977 (1.4%) | 183 (3.4%) | 1055 (3.0%) | 103 (3.8%) | 571 (3.4%) | 1942 (32.9%) | 11890 (9.4%) |
| HF | FU (patient-years) | 2434142 | 1502725 | 18891 | 180677 | 14402 | 84146 | 31162 | 591668 |
|  | n (n/N) | 53481 (11.3%) | 11688 (4.2%) | 2194 (41.3%) | 4152 (11.9%) | 389 (14.3%) | 1678 (9.8%) | 797 (13.5%) | 30620 (24.2%) |
| CKD | FU (patient-years) | 2351938 | 1480781 | 21572 | 150419 | 14239 | 81647 | 29984 | 563358 |
|  | n (n/N) | 93962 (19.8%) | 25766 (9.3%) | 1528 (28.8%) | 15179 (43.3%) | 522 (19.2%) | 3419 (20.1%) | 1475 (25.0%) | 43788 (34.6%) |
| MARCE | FU (patient-years) | 2162778 | 1428267 | 17358 | 143220 | 10945 | 73074 | 23718 | 457896 |
|  | n (n/N) | 159363 (33.7%) | 45757 (16.5%) | 3077 (57.9%) | 17998 (51.4%) | 1553 (57.1%) | 7083 (41.6%) | 3268 (55.4%) | 77711 (61.4%) |

Additional File 1: Table S9. Total event rates and follow up time in in type 2 diabetes by baseline CVRD status
